# Continuous Theta-Burst Stimulation Promotes Paravascular CSF-Interstitial Fluid Exchange through Regulation of Aquaporin-4 Polarization in APP/PS1 Mice

**DOI:** 10.1155/2022/2140524

**Published:** 2022-08-19

**Authors:** Cheng Wu, Tuo Lin, Qian Ding, Ni Zhang, Zi-tong Ou, Gui-yuan Cai, Hong-ying Chen, Jia-yue Xu, Ge Li, Zhong Pei, Guang-qing Xu, Yue Lan

**Affiliations:** ^1^Department of Rehabilitation Medicine, The Second Affiliated Hospital, School of Medicine, South China University of Technology, Guangzhou, China; ^2^Department of Rehabilitation Medicine, Guangdong Provincial People's Hospital, Guangdong Academy of Medical Sciences, Guangzhou 510080, China; ^3^Guangdong Laboratory Animals Monitoring Institute, Guangzhou, China; ^4^Guangdong Key Laboratory for Diagnosis and Treatment of Major Neurological Diseases, The First Affiliated Hospital, Sun Yat-sen University, Guangzhou, China

## Abstract

Amyloid-*β* (A*β*) deposition plays a crucial role in the occurrence and development of Alzheimer's disease (AD), and impaired A*β* clearance is the leading cause of A*β* deposition. Recently, studies have found that the glymphatic system performs similar functions to the peripheral lymphatic system. Glymphatic fluid transport mainly consists of cerebrospinal fluid (CSF) entering the brain from the paravascular space (PVS) by penetrating arteries and CSF and interstitial fluid exchanging mediated by aquaporin-4 (AQP4). This system promotes the drainage of interstitial fluid (ISF) in the parenchyma and removes metabolic waste, including A*β*, in the brain. Glymphatic system dysfunction plays an essential role in the occurrence and progression of AD. Regulation of glymphatic fluid transport may be a critical target for AD therapy. This study explored the regulatory effects of continuous theta-burst stimulation (CTBS) on the glymphatic system in APPswe/PS1dE9 (APP/PS1) mice with two-photon imaging. The results demonstrated that CTBS could increase glymphatic fluid transport, especially CSF and ISF exchange, mediated by improved AQP4 polarization. In addition, the accelerated glymphatic pathway reduced A*β* deposition and enhanced spatial memory cognition. It provided new insight into the clinical prevention and treatment of A*β* deposition-related diseases.

## 1. Introduction

Impaired amyloid-*β* (A*β*) clearance is the leading cause of A*β* deposition in Alzheimer's disease (AD) and causes a series of pathological changes [1–3]. A*β* accumulates in humans several decades before symptoms of AD appear [4, 5]. Blood-brain barrier mediates A*β* clearance through several receptors and transporters, such as the low-density lipoprotein receptor-related protein 1 (LRP1), P-glycoprotein (P-gp), and the receptor for advanced glycation end products (RAGE) [6, 7]. However, promoting blood-brain barrier permeability is dangerous. Recent studies found that the glymphatic system is also an essential pathway for A*β* clearance in the brain [8]. Impairment of the glymphatic system in AD can lead to amyloid plaque formation and cerebral amyloid angiopathy [9]. The timely and effective promotion of the glymphatic system may possess therapeutic effects against AD.

Potential factors affecting the clearance efficiency of the glymphatic system include molecular size, aquaporin-4 (AQP4) expression, localization, and [10]. AQP4 is mainly distributed in astrocytes, ependyma, and choroid plexus in the brain [11]. It can promote cerebrospinal fluid (CSF) drainage from the paravascular space (PVS) to brain interstitial fluid (ISF). Under normal circumstances, these water channels are highly polarized and distributed to the perivascular terminals and encapsulate cerebral blood vessels [12, 13], which plays a vital role in maintaining the normal function of the glymphatic system. About 70% reduction in ISF clearance and 55%–65% reduction in A*β* clearance was observed in AQP4 knockout mice [8, 12, 13]. Therefore, AQP4-dependent glymphatic pathway played a key role in promoting the clearance of soluble A*β* from CSF and extracellular fluid. The expression and localization of AQP4 are closely related to AD patients' pathological changes and functional status [9]. The loss of perivascular AQP4 localization may be a severe predisposing factor in the accumulation of A*β* in AD [9, 14].

Previous studies have found that repetitive transcranial magnetic stimulation (rTMS) can significantly improve cognitive function and memory function and improve depression and anxiety in AD patients [15]. Continuous theta-burst stimulation (CTBS), as a novel rTMS model, is a crucial brain regulation technology [16]. CTBS's rapid regulation of cortical excitability and its relatively long-lasting aftereffects make it practical to regulate brain function [17]. In this study, we intended to explore the therapeutic effect of CTBS in APPswe/PS1dE9 (APP/PS1) mice, focusing on the glymphatic pathway. We believe it would be noteworthy for preventing and treating metabolic waste accumulation-related diseases.

## 2. Materials and Methods

### 2.1. Mouse Strains and Housing

APP/PS1 mice (Twelve males 7–8 months old) on C57BL/6 J background were purchased from Guangdong Medical Experimental Animal Center. Keep all animals in a temperature- and humidity-controlled room under a 12-h/12-h light/dark cycle. We selected the sample size according to previously published reports [18]. Perform the experimental procedures by the guidelines imposed by the Sun Yat-sen University Committee on the Care and Use of Animals (Ethics number: 2018000342). In all experiments, anesthetized animals with 1% pentobarbital (50 mg/kg).

### 2.2. Reagents and Antibodies

Fluorescein isothiocyanate-dextran 70 kDa (FITC, Sigma-Aldrich, FD4; St. Louis, MO, USA) was used to trace CSF movement and rhodamine B-dextran 70 kDa (Sigma-Aldrich, R9379, USA) to label vasculature. All reagents were dissolved in artificial CSF (ACSF) at a concentration of 1%. Antibodies for AQP4 (Alomone Labs, 300-314; Jerusalem, Israel) and glial fibrillary acidic protein (GFAP, Sigma, G3893, USA) were used to detect aquaporins and astrocytes. All secondary antibodies were purchased from Cell Signaling Technology (4408, 4409, 4412, 4413; Carlsbad, CA, USA).

### 2.3. CTBS Administration

**Figure 1:**
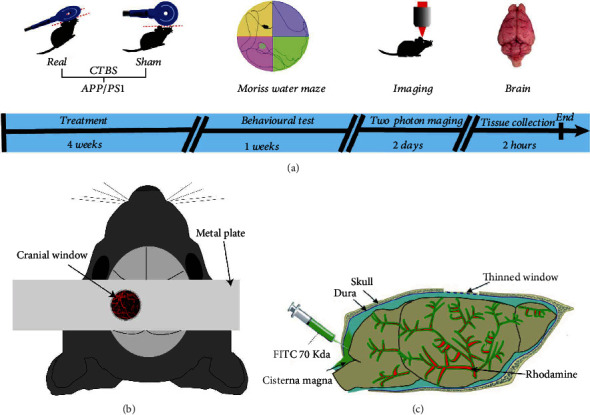
Study protocol. (a) Experimental timeline. Timeline showing a time series event of mouse treatment. APP/PS1 mice were first subjected to continuous theta-burst stimulation (CTBS; Real, AR; Sham, AS) for four weeks, and then the spatial cognition function was tested in the Morris water maze (MWM). Two-photon imaging was used to detect the function of the glymphatic system within two days after the MWM was completed, and the sample tissue was collected within 2 hours after the imaging was completed. (b) A schematic diagram of the mouse cranial window. A thinned cranial window was prepared and fixed on a metal plate. (c). Schematic diagram of Cisterna magna injection. After the cranial window was prepared, rhodamine was injected into the tail vein to label the blood vessels, and then FITC was injected through cisterna magna to prepare for imaging.

CTBS was performed with a Rui Chi magnetic stimulator (Yiruide CCY-IA, Wuhan, China), whose maximum output was 3.0 T. The stimulating coil was a circular coil (external loop diameter of 70 mm) that stimulated the whole brain of the mice. Classical CTBS consists of a continuous 40-s pulse train with a frequency of 50 Hz in the cluster and 5 Hz between sets. Randomly divide twelve APP/PS1 mice into real CTBS group (AR) and sham-CTBS group (AS). The mice were restrained in plastic body-contour-shaped restraint tubes. A stimulation intensity of 25% of the instrument's maximum output (0.75 T) was used. Our current stimulation protocol was conducted according to previous research [19]. In the AR group, the scalp was kept tangent to the coil, while in the AS group, the stimulation coil was perpendicular to the scalp (Figure 1(a)). The other stimulation parameters were the same between the two groups. The stimulation protocol was performed once a day at the same time of the day (10.00–11.00) for four weeks.

### 2.4. Morris Water Maze Test

The Morris water maze (MWM) test tested the cognitive function of the AS and AR groups according to a previously described protocol [20]. The mice were taken to the Behavioral Laboratory to habituate for at least 30 minutes before starting the test. The water maze was isolated from the surrounding space to reduce interference. Each quadrant of the sidewall had a unique sign, which was convenient for the mice to perceive spatial orientation. The MWM test consisted of 5 days of acquisition and one day of probe trial. The mice performed four trials per day during the training, starting from each of the four quadrants. The goal for the mice was to find a hidden platform (10 cm in diameter) located 1 cm below the water surface in a pool 120 cm in diameter. The water was made opaque with nontoxic white tempera paint. The water temperature was maintained at 24 ± 1°C. The latency to the platform was the time (seconds) required for mice to find and climb onto the platform. Each mouse was allowed to remain on the platform for 20 s and returned to its home cage. If the mouse did not find the platform within 60 s, gently guide them to the platform, where it was allowed to remain for 20 s before returning to its home cage. On day 6, we removed the platform and conducted a single 60 s probe trial. The swim paths were recorded with an overhead video camera and tracked with automated software (San Diego Instruments, CA, USA). The camera recorded the movement of the mice. It analyzed the latency to the platform, the number of crossings to the platform, the mean velocity, and the percentage of time spent in the target quadrant.

### 2.5. Cranial Window Surgery

After APP/PS1 mice were anesthetized, we used a stereotaxic device to locate the point 2 mm posterior and 1.7 mm laterally of the bregma. A high-speed skull drill was used to polish the skull to form a cranial window with a diameter of about 2 mm. A self-made perforated metal plate was fixed to the edge of the cranial window using modified acrylic glue (Figure 1(b)).

### 2.6. Intracisternal Tracer Injection

Mice were anesthetized with an intraperitoneal injection of 1% pentobarbital (50 mg/kg). The skin of the neck was cleaned with 70% ethanol and iodine and then shaved. The ophthalmic solution was placed on the eyes to prevent drying. The head of the mouse was positioned in an iron frame. The posterior atlantooccipital membrane overlying the cisterna magna was surgically exposed. For all CSF tracer experiments, 10 *μ*L of tracer (Hamilton syringe coupled to a 30-gauge needle) was infused into the cisterna magna puncture at 2 *μ*L/min for 5 minutes through a microsyringe (BASi, West Lafayette, IN, USA). Previous studies have shown that this rate and duration of tracer infusion do not cause reflux of subarachnoid CSF back into the ventricular CSF compartment, suggesting that the injection did not influence the physiological direction of CSF bulk flow [8, 21, 22]. After injecting, we left the syringe to prevent leakage.

### 2.7. In Vivo Two-Photon Imaging of Glymphatic Pathway Clearance

The mice were fixed on the stage of a two-photon microscope (Leica, DM6000, Wetzlar, Germany). ACSF was perfused during the whole surgical procedure to moisten the cranial window. Throughout the experiment, body temperatures were kept constant at 36.8°C with a feedback-controlled heating pad (RWD Life Science Company, Shenzhen, China). 0.2 mL rhodamine B-dextran 70 kDa was injected intravenously before imaging to visualize the vasculature. A Leica NA 0.95 and a 25× magnification water immersion objective were used at an excitation wavelength of 800 nm. The cerebral vasculature was initially imaged with 512 × 512-pixel frames from the surface to a depth of 200 *μ*m with two *μ*m z-steps. Tracer movement was detected with dual channels. Imaging panels 100 *μ*m below the cortical surface were selected to analyze tracer movement of the PVS with Leica Lite software. For paravascular and interstitial movement, circular regions of interest (ROI) 25 pixels in diameter were centered on the surrounding penetrating arterioles. Mean pixel intensities within these ROIs were measured at each time of taking pictures.

### 2.8. Immunofluorescence

Immunofluorescence analysis was conducted on 10 *μ*m paraformaldehyde-fixed frozen sections. Slices were blocked for one hour at room temperature with normal goat serum and 0.3% Triton and incubated overnight with the primary antibody at 4°C, followed by the secondary antibody for one hour at room temperature. Slices were stained with 4′,6-Diamidino-2′-phenylindole (DAPI, F6057, Sigma-Aldrich, USA), and immunofluorescence images were observed under a confocal microscope (Leica, DM6000, Wetzlar, Germany).

### 2.9. Analysis of AQP4 Expression and Polarization

AQP4 immunofluorescence intensity within each region of the cortex and hippocampus was measured to evaluate global AQP4 expression levels. Astrocytic AQP4 polarization was assessed based on previous research [23]. Each AQP4 immunofluorescence sections were analyzed at two sets of color threshold levels (high and low) in each image. The low stringency region refers to the entire area where AQP4 is expressed, while the high stringency region refers to the place where AQP4 is presented in the perivascular terminal foot. The polarity of AQP4 is the ratio of the low stringency region to the high stringency region. The higher the percentage, the stronger the polarity.

### 2.10. Statistical Analysis and Reproducibility

Data were analyzed with SPSS 20.0 and GraphPad Prism 6.0 and presented as the mean ± SD. Different treatment groups were evaluated using one-way ANOVA with LSD or two-way ANOVA with Tukey's test for multiple comparisons to determine differences among individual groups. The unpaired *t*-test was used when comparing two separate groups. A probability of *P* < 0.05 was indicative of a significant difference between groups. Regardless of the method used, the results were equivalent in magnitude and statistically significant.

## 3. Results

### 3.1. CTBS Improved Spatial Memory in APP/PS1 Mice

**Figure 2:**
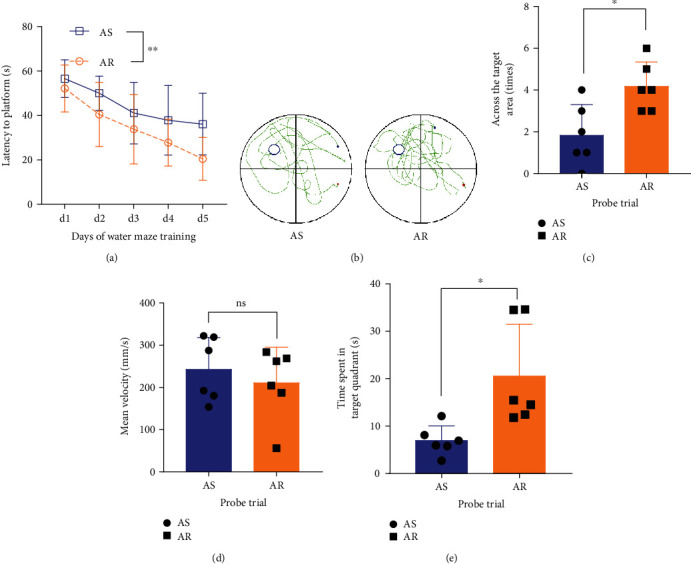
Morris water maze test of spatial cognitive function. (a) Statistical analysis of the latency to reach the platform during the MWM training in the AS and AR groups. (b) Representative swim paths of the mice in the AS and AR groups. (c) Statistical analysis of the number of times crossing the target platform in the AS and AR groups. (d) Statistical analysis of the mean velocity during exploration in the AS and AR groups. (e) Statistical analysis of the percentage of time spent in the target quadrant by the AS and AR groups. Data are presented as the mean ± SD (*n* = 6 mice per group, two-way ANOVA with Tukey's test for multiple comparisons, unpaired *t*-test for comparing two individual groups; ^∗^*P* < 0.05, ^∗∗^*P* < 0.01, ^∗∗∗^*P* < 0.001, and ^∗∗∗∗^*P* < 0.0001).

After four consecutive weeks of stimulation, we tested the effect of CTBS on the cognitive function of APP/PS1 mice by MWM. During the seven days of training, there was a statistically significant difference in the latency to reach the platform between the two groups (AS vs. AR, two-way ANOVA, *F* = 7.86, *P* < 0.001 for time, *F* = 12.08, *P* < 0.01 for group; Figure 2(a)). On day 6 of the probe trial, the crossing number significantly increased in the AR group compared to AS group (AS vs. AR, two-sample *t*-test, *t* = 3.04, *P* < 0.05; Figures 2(b) and 2(c)). The time spent in the target quadrant significantly increased in the AR group compared to the AS group (AS vs. AR, two-sample *t*-test, *t* = 2.935, *P* < 0.05; Figure 2(e)). No significant difference was found in the mean swimming velocity between AS and AR groups (Figure 2(d)). The results demonstrated that the cognitive function of APP/PS1 mice was significantly improved after four weeks of CTBS.

### 3.2. CTBS Accelerates the Glymphatic Clearance Pathway

**Figure 3:**
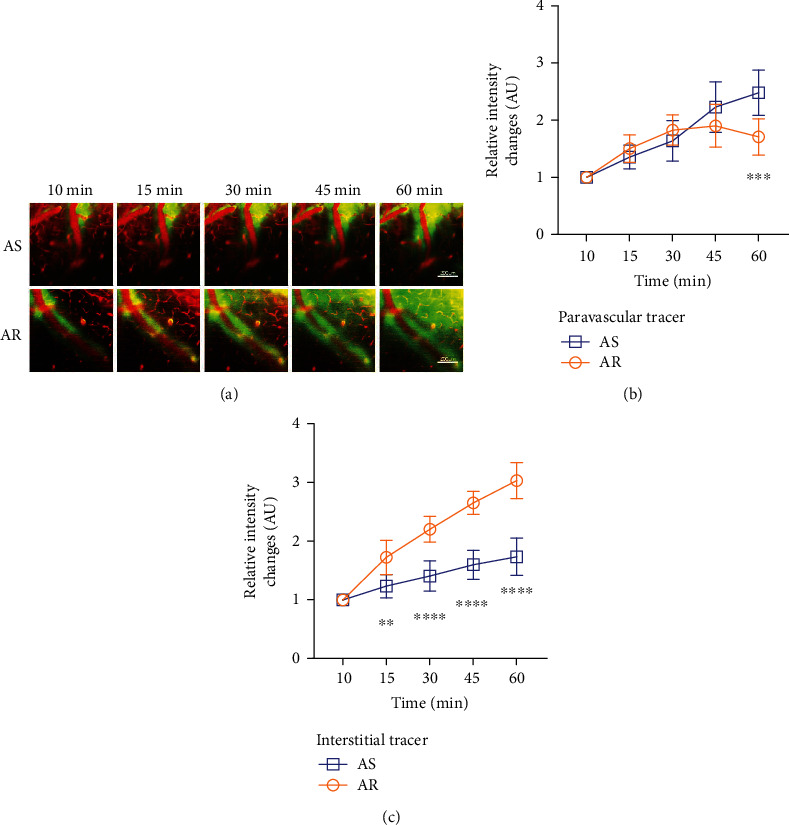
CTBS promotes glymphatic clearance in APP/PS1 mice. (a) In vivo two-photon imaging of tracer clearance through the glymphatic pathway in 10 min, 15 min, 30 min, 45 min, and 60 min in AS and AR groups (250×, scale bar: 200 *μ*m). (b) Statistical quantification of CSF tracer influx into the paravascular space. (c) Statistical quantification of CSF tracer penetration into the surrounding parenchyma. Data are presented as the mean ± SD (*n* = 6 mice per group, two-way ANOVA with Tukey's test for multiple comparisons; ^∗^*P* < 0.05, ^∗∗^*P* < 0.01, ^∗∗∗^*P* < 0.001, and ^∗∗∗∗^*P* < 0.0001).

After rhodamine was injected into the tail vein to label the blood vessels, FITC was injected into the cisterna magna to trace the CSF drainage. Two-photon *in vivo* imaging was used to further observe the effect of CTBS on glymphatic fluid transport in APP/PS1 mice (Figure 3(a)). The results showed that CTBS promoted CSF tracer influx into the PVS and penetration into the ISF in AR group compared to AS group (two-way ANOVA, for paravascular tracer, *F* = 11.84, *P* < 0.01; for interstitial tracer; for interaction factor, *F* = 17.85, *P* < 0.01; Figures 3(b) and 3(c)).

### 3.3. CTBS Reduces A*β* Deposition in the Brain of APP/PS1 Mice

**Figure 4:**
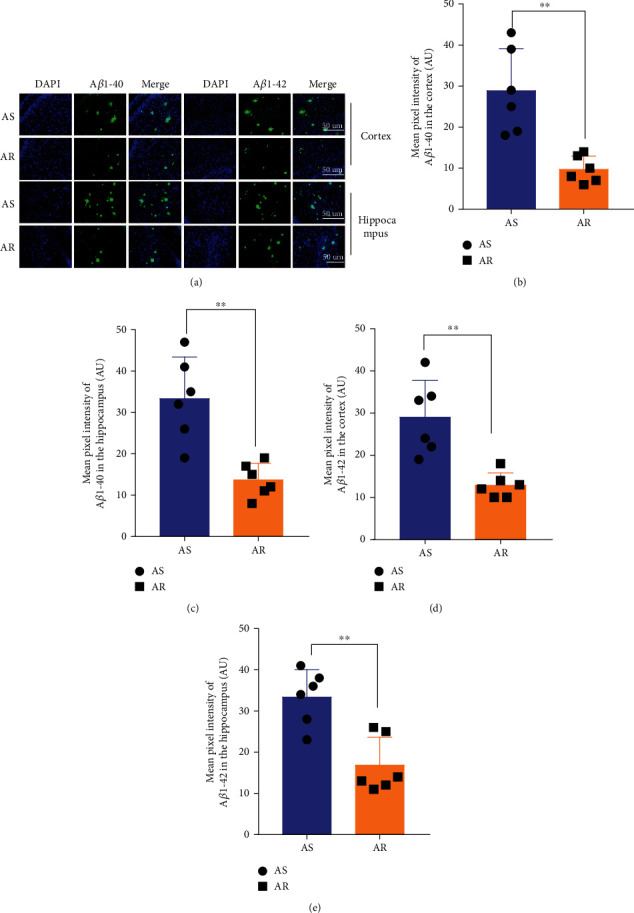
CTBS reduced amyloid *β* deposition in APP/PS1 mice. (a) Immunofluorescence staining of A*β*1-40 or A*β*1-42 in the cortex and hippocampus of AS and AR groups (400×, scale bar: 50 *μ*m). (b and c) Statistical analysis of the mean pixel intensity of A*β*1-40 in the cortex and hippocampus. (d and e) Statistical analysis of the mean pixel intensity of A*β*1-42 in the cortex and hippocampus. Data are presented as the mean ± SD (*n* = 6 mice per group, unpaired *t*-test for comparing two individual groups; ^∗^*P* < 0.05, ^∗∗^*P* < 0.01, ^∗∗∗^*P* < 0.001, and ^∗∗∗∗^*P* < 0.0001).

We conducted immunofluorescence to observe the effect of CTBS on the deposition of A*β* in the brain. Brain sections from the APP/PS1 mice were labeled with A*β*1-40 and A*β*1-42, respectively (Figure 4(a)). The accumulation of A*β* (A*β*1-40/A*β*1-42) in the brain parenchyma (cortex/hippocampus) in the AR group was significantly less than that in the AS group (two-sample *t*-test, for A*β*1-40 in the cortex, *t* = 4.336, *P* < 0.01; for A*β*1-40 in the hippocampus, *t* = 4.425, *P* < 0.001; for A*β*1-42 in the cortex, *t* = 4.276, *P* < 0.001; for A*β*1-42 in the hippocampus, *t* = 4.241, *P* < 0.001; Figures 4(b)–4(e)).

### 3.4. Improved Cognitive Function and Reduced A*β* Deposition in the Brain after CTBS Are Closely Associated with Accelerated Glymphatic Clearance

**Figure 5:**
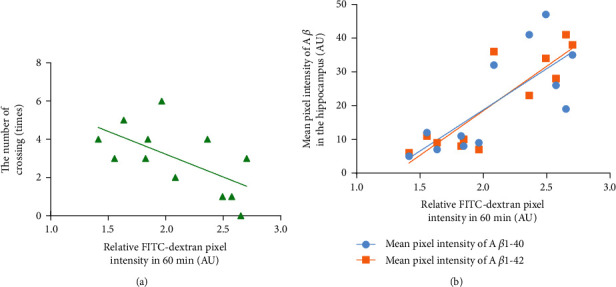
Correlation scatters plot of accelerated glymphatic clearance, improved cognitive function, and reduced A*β* deposition in APP/PS1 mice. (a) Correlation between relative FITC-dextran pixel intensity at 60 min and the number of crossings in the MWM task (*r* = −0.6707, *P* < 0.05). (b) Correlation between relative FITC-dextran pixel intensity at 60 min and the mean pixel intensity of A*β*1-40/A*β*1-42 in the hippocampus (*r* = 0.8671, *P* < 0.01 for A*β*1-40; *r* = 0.7532, *P* < 0.01 for A*β*1-42).

To study the relationship between the improved cognitive function by CTBS and the glymphatic clearance pathway, a correlation analysis between relative FITC-dextran pixel intensity at 60 min and performance in the MWM task (number of crossings) was conducted. Correlation analysis showed that the number of times crossing the platform in the water maze exploration experiment was negatively correlated with the fluorescence intensity in the APP/PS1 mice at 60 min (*r* = −0.6707, *P* < 0.05; Figure 5(a)). The results showed a significant correlation between accelerated glymphatic clearance and improved cognitive function. To explore the relationship between the reduction in A*β* deposition in the brain and the acceleration of the glymphatic clearance pathway, correlations between the relative FITC-dextran pixel intensity at 60 min and A*β* deposition in the brain parenchyma were conducted. The results showed that the amount of A*β* (1-40/1-42) deposited in the brain parenchyma was positively correlated with the fluorescence intensity at 60 min in the APP/PS1 mice (for A*β*1-40, *r* = 0.8671, *P* < 0.01; for A*β*1-42, *r* = 0.7532, *P* < 0.01; Figure 5(b)). The results showed a significant correlation between accelerated glymphatic clearance and decreased deposition of A*β*.

### 3.5. CTBS Promotes the Polarization Distribution of AQP4

**Figure 6:**
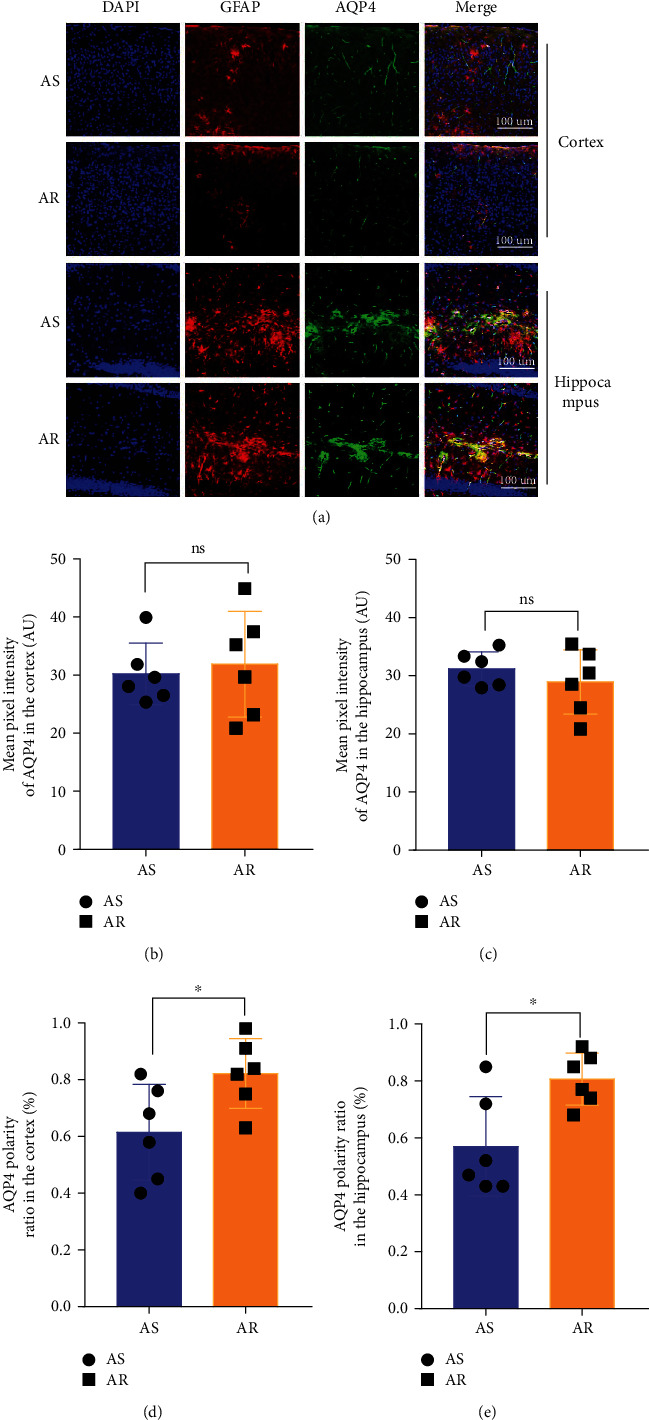
CTBS improved AQP4 polarization in APP/PS1 mice. (a) Immunofluorescent staining of AQP4 in the cortex and hippocampus (200×, scale bar: 100 *μ*m). (b) Statistical analysis of relative AQP4 expression levels in the cortex. (c) Statistical analysis of relative AQP4 expression levels in the hippocampus. (d) Statistical analysis of relative perivascular polarization of AQP4 in the cortex. (e) Statistical analysis of relative perivascular polarization of AQP4 in the hippocampus. Data are presented as the mean ± SD (*n* = 6 mice per group, unpaired *t*-test for comparing two individual groups; ^∗^*P* < 0.05, ^∗∗^*P* < 0.01, ^∗∗∗^*P* < 0.001, ^∗∗∗∗^*P* < 0.0001).

AQP4 is the main pathway for CSF influx from the PVS to the parenchyma. Since CTBS significantly improves cerebral ISF drainage in AR group compared to AS group, immunofluorescence of GFAP and AQP4 was conducted to observe the effect of CTBS on AQP4 polarization and expression (Figure 6(a)). The results showed no significant difference in the expression of AQP4 between AS and AR groups both in the cortex (Figure 6(b)) and hippocampus (Figure 6(c)). At the same time, CTBS significantly improved AQP4 polarization in AR groups both in the cortex (Figure 6(d)) and hippocampus (Figure 6(e)).

Taken together, CTBS improved cognitive function, accelerated the glymphatic pathway, and reduced A*β* deposition in the brain of APP/PS1 mice. The improved cognitive function and reduced A*β* deposition were significantly correlated with accelerated glymphatic drainage.

## 4. Discussion

AD patients already have A*β* deposition ten to several decades before the onset of the disease [24–26], and it is now clear that the glymphatic system is an essential pathway for A*β* clearance in the brain [8, 27]. Therefore, early regulation of glymphatic pathway clearance is beneficial to delaying and preventing disease progression and prolonging healthy life years. In this study, a two-photon in vivo imaging technique was used to investigate the effect and mechanism of CTBS on the drainage of the glymphatic pathway in APP/PS1 mice. We found that CTBS improved the function of the glymphatic pathway in APP/PS1 mice, which was mainly attributed to an improvement in the polarization distribution of AQP4. Accelerated glymphatic clearance reduces A*β* deposition in the brain and improves spatial cognition in APP/PS1 mice, which laid a foundation for the application of CTBS in diseases related to glymphatic system dysfunction in patients.

### 4.1. CTBS Improves Spatial Memory in APP/PS1 Mice

APP/PS1 double transgenic mice overexpress human amyloid precursor protein (APPsw) and mutant forms of presenilin 1 (m146L) genes, which are characterized by early amyloid deposition and early cognitive impairment and glymphatic dysfunction [28]. RTMS has been shown to improve cognitive impairment in AD patients [29, 30]. Since CTBS is a more efficient method of repetitive transcranial magnetic stimulation, we further investigated the effect of CTBS on cognitive function in APP/PS1 mice. The MWM test was conducted after four weeks of real/sham CTBS to APP/PS1 mice. The results showed that real CTBS (AR group) could significantly improve the spatial memory function (Figure 2). Consistent with our study, Choung et al. found that high-frequency rTMS can substantially enhance the spatial memory function of AD mice, which may be related to the activation of the dopaminergic system and the increase of brain-derived neurotrophic factor in the brain [31].

### 4.2. CTBS Promotes Glymphatic Clearance in APP/PS1 Mice

In the glymphatic system, CSF rapidly enters the brain parenchyma along with the PVS of the penetrating arteries, and metabolic wastes are removed from the brain through the exchange between CSF and ISF [8]. A*β* deposition is one of the core mechanisms of cognitive dysfunction in AD patients, and it is closely related to glymphatic dysfunction [25, 32]. The MWM test in this study showed that CTBS could improve cognitive function in APP/PS1 mice, but the specific mechanism was unclear. Therefore, we injected FITC into the cisterna magna to label CSF drainage and injected rhodamine into the tail vein to trace blood flow. Two-photon in vivo imaging was used to observe whether CTBS can promote glymphatic drainage and then exert neuroprotective effects. After 60 minutes of observation, we found that CTBS could significantly accelerate the glymphatic pathway in APP/PS1 mice. The rate of PVS drainage and ISF penetration in the AR group increased. They indicated that improved cognitive function in APP/PS1 mice might be related to the increased glymphatic clearance.

### 4.3. Decreased A*β* Deposition in the Brain Parenchyma after CTBS

To further verify that CTBS accelerates the glymphatic pathway and improves cognitive function related to A*β* deposition, immunofluorescence staining was performed to observe A*β*1-40 and A*β*1-42 in the cortex and hippocampus of AS group and AR group (Figure 3). The results showed that the deposition of A*β*1-40 and A*β*1-42 in mice's cerebral cortex and hippocampus in the AR group was significantly less than that in the AS group after four weeks of CTBS. It shows that CTBS accelerates the glymphatic clearance pathway, and there is indeed a close relationship between the reduction of A*β* deposition and cognitive function improvement. Soluble amyloid oligomers are the primary cause of AD pathogenesis and a clinically proven target for slowing the disease's progression [33]. Soluble A*β* oligomers can produce cytotoxicity, contribute to synaptic deficits, and initiate the detrimental cascade involved in the pathology of AD [34–36]. Therefore, CTBS may improve cognitive function by promoting the clearance of soluble A*β* and reducing A*β* deposition in the brain parenchyma.

### 4.4. CTBS Promotes A*β* Clearance and Improves Spatial Memory Function through the Acceleration of the Glymphatic System

AD is a neurodegenerative disease clinically manifested by a comprehensive decline in cognitive function accompanied by abnormal mental behavior and the eventual loss of activities of daily living. The use of rTMS technology for targeted intervention in AD and its high-risk groups to improve patients' cognitive function and delay the development of dementia has attracted more and more attention.

The mechanisms by which rTMS improves cognitive function in AD patients include modulating the excitability of the cerebral cortex [37, 38], altering cerebral blood flow and glucose metabolism [39], modulating synaptic plasticity and connectivity of neuronal cells, and modulating neurotrophic and neurotransmitters [40]. This study found that CTBS stimulation can accelerate the essential metabolic waste removal pathway in the brain-glymphatic pathway, which may provide a new mechanism pathway for the treatment of AD by TMS.

To examine the relationship between accelerated glymphatic clearance, improved spatial cognition, and reduced A*β* deposition, we analyzed the relationship between glymphatic clearance and cognitive function and the relationship between glymphatic clearance and deposition of A*β*1-42 and A*β*1-40 in the brain. Correlation analysis showed that the 60-minute metabolic residual of the glymphatic system was negatively correlated with the number of crossing platforms in the exploration experiment and positively correlated with the amount of A*β* deposition in the brain parenchyma. It further indicates that CTBS indeed accelerates the glymphatic clearance pathway, promotes A*β* clearance, reduces A*β* deposition in the brain, and improves spatial cognition. Consistent with our findings are the studies by Lin et al. [41] and Liu et al. [19].

### 4.5. CTBS Accelerates Glymphatic Clearance by Improving the Polarized Distribution of AQP4

Through two-photon in vivo imaging, it can be found that CTBS accelerates the glymphatic pathway, which is mainly manifested in the diffusion of CSF from the PVS to the ISF. This process requires AQP4, which is distributed on astrocyte pseudopodia of the vascular basement membrane. AQP4 is a substantial channel for the clearance of A*β* in the glymphatic system, and the change of the polarization state in AQP4 will affect the efficiency of A*β* clearance [42]. Activated astrocytes with altered AQP4 polarization state have been identified in the brain tissue of AD patients and several AD mouse models, seriously affecting the efficiency of glymphatic clearance [43].

To further explore whether CTBS could affect the expression and distribution of AQP4, immunofluorescence staining of astrocytes and AQP4 was performed to observe the expression and distribution of AQP4. The study found that CTBS had no obvious effect on the expression of AQP4 but improved the polarized distribution of AQP4 in the cortex and hippocampus of APP/PS1 mice. These results suggest that CTBS accelerates the glymphatic pathway by enhancing the polarization state distribution of AQP4. Consistent with our findings, Liu et al. [19] found that CTBS accelerated PVS drainage and ISF diffusion in sleep-deprived rats and restored the polarity loss of AQP4 in sleep-deprived rats.

The expression and distribution of AQP4 are closely related to protein phosphorylation, *α*-syntrophin, and agrin [44–46]. Studies have reported that *γ*-aminobutyric acid A receptor (GABAAR) signaling can promote the expression of AQP4 by reducing water channel-related serine phosphorylation and promoting water exchange in the subependymal zone (SEZ) [15]. The study by Li et al. suggested that CTBS may increase the inhibitory activity of interneurons, resulting in elevated GABA concentrations [47]. In this study, CTBS may regulate the polarization state of AQP4 through the GABA pathway, and the specific mechanism remains to be further explored later.

In summary, our study found that CTBS can improve the polarization state of AQP4 in APP/PS1 mice, accelerate the glymphatic clearance pathway, thereby reducing A*β* deposition in the brain parenchyma, and improve the cognitive function of APP/PS1 mice. This study also has certain limitations. First, glymphatic system clearance is an orderly overall process. Due to the limitations of technical means, we can only observe the overall effect of PVS drainage and ISF diffusion clearance at different time points after CTBS and conduct a comprehensive assessment. Secondly, due to the limitations of stimulation methods, we used whole-brain stimulation for mice, which lacks precision compared to the inspiration of fixed sites when TMS is applied to the patient. It is expected that more focal coils with greater stimulation intensity will be developed in the future.

## 5. Conclusion

AD is one of the frequently occurring diseases in the elderly population. Early detection, early diagnosis, and early treatment are of great significance for controlling pathological processes and promoting the recovery of the disease. The intervention of the glymphatic pathway through CTBS was beneficial for accelerating the clearance of A*β* in the brain and improving the pathological damage and clinical cognitive dysfunction of glymphatic dysfunction-related diseases.

## Data Availability

The data is available and reproducible in our study.

## Abbreviations

A*β*: Amyloid-*β* peptide
AD: Alzheimer's disease
GFAP: Glial fibrillary acidic protein
AQP4: Aquaporin-4
CSF: Cerebrospinal fluid
ACSF: Artificial cerebrospinal fluid
PVS: Paravascular space
ISF: Interstitial fluid
rTMS: Repetitive transcranial magnetic stimulation
APP/PS1: APPswe/PS1dE9
FITC: Fluorescein isothiocyanate
DAPI: 4′,6-Diamidino-2′-phenylindole
CTBS: Continuous theta-burst stimulation
AR: APP/PS1 with real CTBS
AS: APP/PS1 with sham CTBS
MWM: Morris water maze test
GABA: *γ*-Aminobutyric acid.
